# NDDVD: an integrated and manually curated Neurodegenerative Diseases Variation Database

**DOI:** 10.1093/database/bay018

**Published:** 2018-03-05

**Authors:** Yang Yang, Chen Xu, Xingyun Liu, Chao Xu, Yuanyuan Zhang, Li Shen, Mauno Vihinen, Bairong Shen

**Affiliations:** 1 Center for Systems Biology, Soochow University, No1. Shizi Street, Suzhou, Jiangsu 215006, China; 2 School of Computer Science and Technology, Soochow University, No1. Shizi Street, Suzhou, Jiangsu 215006, China; 3 Department of Experimental Medical Science, Lund University, SE-221 84 Lund, Sweden and; 4 Department of Genetics and Systems Biology Institute, Yale University School of Medicine, West Haven, CT 06516, USA

## Abstract

Neurodegenerative diseases (NDDs) are associated with genetic variations including point substitutions, copy number alterations, insertions and deletions. At present, a few genetic variation repositories for some individual NDDs have been created, however, these databases are needed to be integrated and expanded to all the NDDs for systems biological investigation. We here build a relational database termed as NDDVD to integrate all the variations of NDDs using Leiden Open Variation Database (LOVD) platform. The items in the NDDVD are collected manually from PubMed or extracted from the existed variation databases. The cross-disease database includes over 6374 genetic variations of 289 genes associated with 37 different NDDs. The patterns, conservations and biological functions for variations in different NDDs are statistically compared and a user-friendly interface is provided for NDDVD at: http://bioinf.suda.edu.cn/NDDvarbase/LOVDv.3.0.

**URL**: http://bioinf.suda.edu.cn/NDDvarbase/LOVDv.3.0

## Introduction

Most of diseases are associated with genetic variations including point substitutions, copy number alterations, insertions and deletions. The genetic variations in DNA sequences may lead to abnormal messenger RNA splicing or coding and produce pathogenic proteins. It is well-known that the relationship between genes and diseases are often multiple to multiple mode i.e. one disease is often associated with many variations in different genes; the variations in the same gene may be responsible for several different diseases (1). These disease-related genetic variations were uncovered and kept in many individual studies. The literature-based genetic variation repository, therefore, exerts considerable significance in systematically pathology study.

Neurodegenerative diseases (NDDs), caused by the progressive dysfunction of neurons, are very common worldwide, affecting people of all ages but especially the aged ones. Since the first patient was diagnosed with Alzheimer’s disease a century ago (2), millions have been found suffering from the neurodegenerative disorders such as Alzheimer’s, Parkinson’s and Amyotrophic lateral sclerosis. According to the MeSH database (http://www.ncbi.nlm.nih.gov/mesh), there are totally 55 subdivisions under the term ‘neurodegenerative disease.’ Although the pathogenesis of NDDs can be quite different, a growing number of studies implied that many NDDs are associated with genetic variations, most of which locate in completely unrelated genes. In spite of the separate symptoms, the intracellular mechanisms of different NDDs share a lot in common. Mitochondrial dysfunction and oxidative stress were reported to play a causal role in pathogenesis for many NDDs (3). The genetic deficiencies in different disorders are also associated, for instance, the poly glutamine mutant which is induced by repeat of CAG nucleotide triplet, was regarded as the dominant pathogenesis for many inherited NDDs such as Huntington’s disease and spinocerebellar ataxias (4).

An integrated literature-based variation database for a series of related diseases can serve as a research platform for further discovering of relationships between diseases and genetic variations. The variation data of NDDs can be compared with that of other diseases, e.g. immunodeficiencies (5), to find their similarities and differences. It can provide data for identification of common variation spectrum within NDDs and for developing universal biomarkers or drugs for NDDs as well. At present, 4 Locus-Specific Databases (LSDBs) have been created for 5 individual NDDs (Table 1). However, information of more other NDDs is not collected yet. In this study, we collected variation data manually for all NDDs and stored them in the latest LOVD system (6). We intended to provide a complete and cross-diseases platform including up-to-date genetic variation information related to all subdivisions of NDDs. The integrated database will serve as a valuable tool for quickly querying of the NDD-related variations and systematically analysing of the relationships between diseases and variations.

**Table 1.** Existing LSDBs for NDDs

No.	Disease name	Database name	Website address
1	Alzheimer disease/Frontotemporal lobar degeneration	Alzheimer disease and frontotemporal dementia mutation database (24)	http://www.molgen.ua.ac.be/ADMutations/
2	Alzheimer disease/frontotemporal lobar degeneration	Alzforum mutation database (25)	http://www.alzforum.org/mutations
3	Amyotrophic lateral sclerosis (ALS)	ALS mutation database (26)	https://gwas.biosciencedbc.jp/cgi-bin/hvdb/hv_pos.cgi? gid=EG104
4	Amyotrophic lateral sclerosis (ALS)	ALSOD (27)	http://alsod.iop.kcl.ac.uk/Als/Overview/gene.aspx? gene_id=SOD1
5	Parkinson disease	Parkinson disease mutation database (24)	http://www.molgen.ua.ac.be/PDmutDB/
6	Parkinson disease	Parkinson disease mutation database	https://gwas.biosciencedbc.jp/cgi-bin/hvdb/hv_disease.cgi? did=2
7	Parkinson disease	Parkinson’s disease mutation database	http://grenada.lumc.nl/LOVD2/TPI/home.php
8	Rett syndrome	RettBASE (28)	http://mecp2.chw.edu.au/#mutations
9	Tuberous sclerosis	Tuberous sclerosis database	http://chromium.lovd.nl/LOVD2/TSC/home.php
10	Tuberous sclerosis	BIPMed–variants in tuberous sclerosis patients from Brazil (29)	http://bipmed.iqm.unicamp.br/tuberous-sclerosis/genes/TSC2

## Materials and methods

### Data source

The disease list of NDDs was obtained from the category of ‘neurodegenerative disease’ in MeSH database (http://www.ncbi.nlm.nih.gov/mesh). Each disease associated gene and variation was collected by the following method, i.e. the disease name or aliases plus ‘variation’ or ‘mutation’ was used as the keyword for querying in PubMed. The disease aliases are listed in Supplementary Table S1. The query results were further checked and the disease-related genes, SNPs or amino acid variations were then extracted manually. The variations collected encompass both those associated with autosomal dominant disease as well as those identified through association studies which affect disease risk. Variants in both translated and un-translated regions are collected. The general gene information such as gene synonym and genome location was fetched from NCBI. For variants with SNP IDs, the associated genes are described as the same as that used in dbSNP. If a variant does not located exactly within a gene region in dbSNP, we describe it based on their genomic information in NDDVD. The personalized information of patients, especially their ethnicities, demographic and epidemiological data if have, was collected from the reference papers to provide data for the future stratified medicine study, since these data will be useful to the future classification of patients to different subgroups (Supplementary Table S2). We followed the guidelines and standard for establishing Locus Specific Databases (7) when building NDDVD. That is to say, HGNC gene names (8) and HGVS variation nomenclature were applied when we built our database (9). For each gene, the reference mRNA and amino acid sequences were searched and recorded from locus reference genomic (LRG) database (10). If the sequence was not available, we used RefSeq and UniProt instead. The biological effects of DNA, RNA and protein variations were annotated using Variation Ontology (VariO) (11). The steps of this manually screening process are shown in Supplementary Figure S1.

### LOVD 3.0

LOVD (Leiden Open Variation Database) platform, supplied by Leiden University Medical Center, provides a flexible, freely available tool for gene-centered collection and display of DNA variations. The current version LOVD 3.0 extends this idea to provide storage for patient-centered data, NGS data and even variants outside of genes. The NDDVD database is established based on this new LOVD version which supports the storing of genes and variations of different diseases in one database.

## Results and discussion

### Web service architecture and user interface

In LOVD3.0 view, the information is shown in eight different tags: genes, transcripts, variants, individuals, diseases, screenings, submit and documentation. It is a LOVD standard structure, which provides series useful information through a user-friendly interface by clicking each hyperlink. Users can also register as submitters to search the database or submit new genes and variations. The LOVD also supplies functions for importing and exporting data between different resources and the NDDVD is available at http://bioinf.suda.edu.cn/NDDvarbase/LOVDv.3.0.

### Variation statistics

The original disease list of neurodegenerative disease from MeSH database contains 55 subdivisions. Several diseases are counted more than once since they are in different classifications, such as, ‘Gerstmann-Straussler-Scheinker Disease’ existed in both ‘Heredodegenerative Disorders, Nervous System’ and ‘Prion Diseases’ groups; ‘Shy-Drager Syndrome’ were classified to ‘Multiple System Atrophy’ and ‘Shy-Drager Syndrome’ groups, *etc*. In addition, no variation record reported for nine diseases in PubMed yet, the nice diseases are Lambert-Eaton myasthenic syndrome, limbic encephalitis, myelitis, transverse, opsoclonus-myoclonus syndrome, paraneoplastic cerebellar degeneration, paraneoplastic polyneuropathy, postpoliomyelitis syndrome, subacute combined degeneration and diffuse neurofibrillary tangles with calcification. There are some data associated with Huntington disease, myotonic dystrophy and Olivopontocerebellar Atrophies, but these data are not suitable for LOVD 3.0, we therefore have a final list of 37 diseases (Table 2). Up to now 1942 PubMed citations were manually screened, checked and 6374 related DNA variations for 289 genes were extracted and stored in our database. The GO analysis of these genes was done and the result is shown in Supplementary Material.

**Table 2.** Neurodegenerative disease associated genes and variation collected in LOVD 3.0

No.	Disease name	Associated genes	No. of variations	No. of references
1	Alzheimer disease	ABCA7, ABCB1, ADRA1A, AGBL3, ANKS1B, APOE, APP, ATP8B3, BCL3, BIN1, C16orf96, C1orf112, C3orf20, CASS4, CD2AP, CD33, CELF1, CELF2, CENPJ, CFAP70, CHGB, CHMP2B, CHRNB2, CLU, CR1, CSMD1, CST3, CTSF, DSG2, EBLN1, EPHA1-AS1, EXOC3L2, FAM47E, FANCD2, FERMT2, FPR1, FRAS1, FRMD4A, GAL3ST4, GPR45, GRIN2B, HERC6, HFE, HMGCR, IL1B, INPP5D, IP6K3, IPMK, IQCK, KCNQ3, KIF13B, KLHDC4, LRAT, MAGI3, MAPT, MEF2C-AS1, MS4A1, MS4A13, MS4A14, MS4A2, MS4A3, MS4A4A, MS4A4E, MS4A6A, MS4A6E, MS4A7, MSRB3, MYCBPAP, NECTIN2, NFATC1, NFIC, NLGN1, NT5C3A, OPRD1, OPRM1, OR52E4, PDE6B, PEBP4, PICALM, PRNP, PSAP, PSEN1, PSEN2, PTK2B, PVR, QRFPR, RGS11, SIRT1, SLC22A14, SLC24A4, SORCS1, SORL1, SPI1, SUN2, SYNPR, TFAM, TM2D3, TNK1, TOMM40, TP53INP1, TREM2, TREML1, TREML2, TREML4, TTBK2, TTR, UNC5C, WDR46, ZCWPW1, ZNF646	824	219
2	Alexander disease	GFAP	108	58
3	Amyotrophic lateral sclerosis	ALS2, ANG, APEX1, ARHGEF28, C9orf72, CCNF, CHCHD10, CHGB, CHMP2B, DAO, DCTN1, FUS, GLE1, GRN, HFE, HNRNPA1, KIF5A, LIF, LRSAM1, MATR3, MOB3B, OGG1, OPTN, PARK7, PFN1, PON1, PON2, PRPH, SETX, SIGMAR1, SOD1, SPAST, SQSTM1, SS18L1, TARDBP, TBK1, TUBA4A, UBQLN2, UNC13A, VAPB, VCP	762	283
4	Canavan disease	ASPA	86	28
5	Cockayne syndrome	ERCC5, ERCC6, ERCC8	124	21
6	Creutzfeldt-Jakob disease	PRNP, SPRN	70	54
7	Dystonia musculorum deformans	ADCY5, ATM, ATP1A3, GCH1, GNAL, PNKD, PRKRA, SGCE, SLC2A1, THAP1, TOR1A	173	90
8	Familial amyloid neuropathies	APOA1, GSN, TTR	147	110
9	Fatal familial insomnia	PRNP	8	8
10	Frontotemporal lobar degeneration	CCNF, CHCHD10, CHMP2B, DAPK1, FUS, GFAP, GRN, GSK3B, LRRK2, MAPT, MOB3B, OPTN, PRNP, PSEN1, SOD1, SQSTM1, TARDBP, TBK1, TMEM106B, TREM2, UBQLN2, VCP	376	230
11	Gerstmann-Straussler-Scheinker disease	PRNP	36	29
12	Hepatolenticular degeneration	ATP7B	166	51
13	Hereditary sensory and autonomic neuropathy	FAM134B, IKBKAP, NGF, NTRK1, PRNP, RAB7A, SPTLC1, SPTLC2, WNK1	88	33
14	Hereditary sensory and motor neuropathy	DCAF8, DYNC1H1, EGR2, FGD4, FIG4, GDAP1, GJB1, HSPB2, HSPB8, KIF1B, LITAF, LMNA, MFN2, MPZ, MTMR2, NDRG1, NEFL, PEX7, PHYH, PMP22, PRX, RAB7A, SBF2, SH3TC2, SLC12A6, TFG	248	140
15	Kuru	PRNP	1	1
16	Lewy body dementia	CYP2D6, DNAJC13, GBA, LRRK2, PRNP, PSEN1, PSEN2, SNCA, SNCB	32	12
17	Lafora disease	EPM2A, NHLRC1	119	25
18	Lambert-Eaton myasthenic syndrome	SYT2	2	1
19	Lesch-Nyhan syndrome	HPRT1	173	55
20	Myotonia congenita	CLCN1, SCN4A	117	55
21	Menkes Kinky hair syndrome	ATP7A	163	26
22	Multiple system atrophy	COQ2, POLG	26	5
23	Neuronal ceroid-lipofuscinoses	CLCN6, CLN3, CLN5, CLN6, CLN8, CTSD, MFSD8, POLG, PPT1, SGSH, TPP1	393	11
24	NeuroFibromatoses	NF2	109	6
25	Optic atrophy	AFG3L2, MFN2, OPA1, OPA3, SLC25A46	298	61
26	Parkinson disease	ABCA7, ADORA1, APOE, BST1, BTNL2, CD2AP, CLU, CR1, DGKQ, DNAJC13, FBXO7, GAK, GALNT3, GBA, GCH1, HLA-DRA, LRRK2, MAPT, MS4A6A, NUCKS1, PARK2, PARK7, PCGF3, PICALM, PINK1, PM20D1, PODXL, PRDM2, PRNP, PTRHD1, RIC3, RIT2, SEMA5A, SLC2A13, SLC41A1, SLC45A3, SLC50A1, SNCA, SPPL2C, SREBF1, SYNJ1, TMEM175, VPS35	616	78
27	Pantothenate kinase-associated neurodegeneration	PANK2, RAB39B	132	31
28	Pelizaeus–Merzbacher disease	PLP1	96	49
29	Progressive Bulbar palsy	SOD1, TTR	3	3
30	Progressive supranuclear palsy	DCTN1, MAPT, PARK2	16	10
31	Rett syndrome	CDKL5, FOXG1, MECP2	394	100
32	Spinocerebellar degenerations	AFG3L2, C10orf2, CACNA1A, CACNA1G, ELOVL4, ELOVL5, ITPR1, KCNC3, KCND3, SPTBN2, TGM6, TMEM240, TTBK2	53	33
33	Spinal muscular atrophy of adults	HEXA, LMNA, SMN1, VAPB	41	17
34	Spinal muscular atrophies of childhood	HEXA, IGHMBP2, SMN1	49	22
35	Tourette syndrome	HDC, SLITRK1	6	3
36	Tuberous sclerosis	TSC1, TSC2	575	22
37	Unverricht-Lundborg syndrome	CSTB, PRICKLE1, SCARB2	19	14

**Figure 1.**
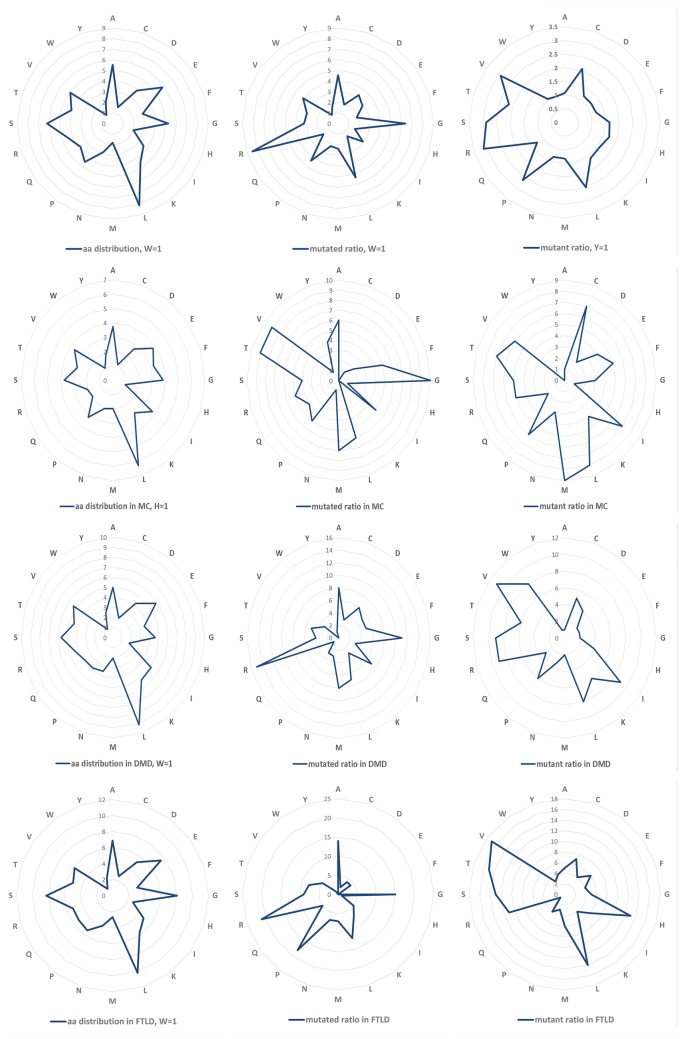
Amino Acid distribution and variation profiles. Top row, amino acid distribution (left), overall mutability of mutated (middle) and mutant residues (right) for all the NDDs related proteins. The same information for Myotonia Congenita (MC), Dystonia Musculorum Deformans (DMD) and Hereditary Sensory and Frontotemporal Lobar Degeneration (FTLD) are presented, respectively, in the lower three rows.

Totally, 5680 amino acid variations were collected and 2839 of them are substitutions without duplicates. Among these variations, the arginine (R) residue is the most common one (both in mutated residues and mutants), which is in agreement with the previous study (12). Top row of Figure 1 shows the amino acid distributions for the wild (left), mutated (middle) and mutant (right) residues of the studied proteins. For the wild amino acids in the studied NDD associated proteins, Tryptophan (W) happened with the lowest observed frequency and was chosen as reference (marked ‘1’). We further calculated the mutability of amino acids as both mutated (middle) and mutant (right) residues. The result shows that R, G, L are the most common mutated residues while R, V, S are highly mutant ones in NDD associated proteins. The variation profiles for all NDDs are similar as that of a larger dataset illustrated in the previous study which contains over 2000 variations related to multiple diseases, as well as some variations related to some NDDs, like amyotrophic lateral sclerosis, etc. (13).

We chose three diseases abundant with variations, i.e. Myotonia Congenita (MC), Dystonia Musculorum Deformans (DMD) as well as Frontotemporal Lobar Degeneration (FTLD) to study their mutation profiles (lower three rows in Figure 1). Arginine (R) is the most common variant residues for all the NDD diseases, the same as analysed in previous study. The previous research reported that random variations at W and C are the most pathogenic (12). The mutant C in MC and the mutant W in DMD are one of the most common mutants in the disease, although this is not observed in MC (W), DMD(C) and FTLD (W and C). This could be caused by the low occurrence rate of C and W residue itself in the disease associated proteins. This difference needs to be further investigated considering their specific pathogenic mechanisms.

**Figure 2.**
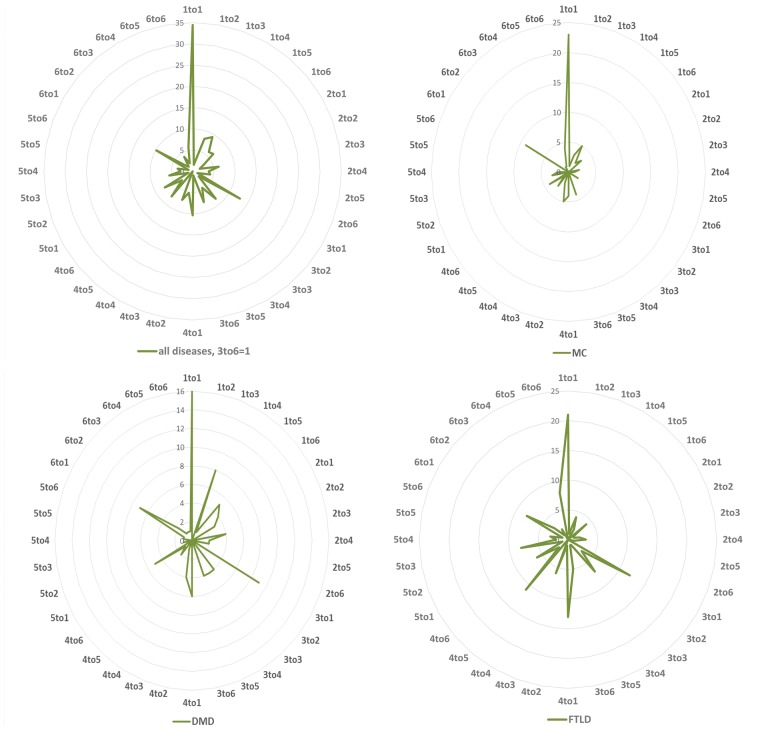
Variation distribution according to physiochemical properties. Left top: mutability distribution for all NDDs in 36 variation situations: number 1–6 denotes to 6 groups of amino acids according to their physiochemical properties: (1) hydrophobic (V, I, L, F, M, W, Y, C), (2) negatively charged (D, E), (3) positively charged (R, K, H), (4) conformational (G, P), (5) polar (N, Q, S) and (6) Alanine and Threonine (A, T) group. The same information for Myotonia Congenita (MC), Dystonia Musculorum Deformans (DMD) and Hereditary Sensory and Frontotemporal Lobar Degeneration (FTLD) are displayed in the right top, left and right bottoms, respectively.

To investigate the functional effects of these variations, we grouped the 20 amino acids into six groups based on their physicochemical properties as, hydrophobic (V, I, L, F, M, W, Y, C), negatively charged (D and E), positively charged (R, K, H), conformational (G and P), polar (N, Q, S) and (A and T) (14). Therefore, the variations (substitutions) can be divided into 36 types based on the changes between 6 types of mutated residues and 6 types of mutants. For all the NDD diseases studied here, the most common mutations are physicochemical property changes from hydrophobic to itself (1 to 1), from positive charged, conformational, A and T to hydrophobic (3 to 1, 4 to 1 and 6 to 1), respectively. The variation profiles are partially similar for MC, DMD and FTLD, e.g. the variation ratios from hydrophobic to hydrophobic residues are very high in all three diseases, and it is easy to be understood since the hydrophobic group is the biggest one among the six groups. There are some obvious different variation profiles between these diseases, e.g. variation from positively charged to hydrophobic (3 to 1) is one of the lowest types for MC, while for DMD and FTLD it is one of the highest types; for FTLD conformational to polar (4 to 5) is very common, as shown in Figure 2.

### Biological function analysis of the NDD associated variations

To study the biological functions of the variations, especially the amino acid substitutions, a number of models and tools were developed to characterize their effects to protein’s sequence conservation, structural stability, aggregation, disorder, etc.(14–17). We developed a SVM classifier for predicting the effects of variations on protein stability based mainly on structural information especially the change of contact energy (17, 18). PON-P2 is a machine learning-based classifier and groups the variants into pathogenic, neutral and unknown classes, on the basis of random forest probability score (19). SIFT predicts the effects of all possible substitutions at each position in the protein sequence by using sequence homology (20).

We still chose the previous three diseases, MC, DMD and FTLD for the analysing. For MC there are 83 amino acid substitutions collected from 2 genes, SCN4 and CLCN1, corresponding to protein sodium channel protein type 4 subunit alpha and chloride channel protein 1, respectively. The results are shown in Supplementary Table S3. Both proteins have such a long sequence, and the results indicated that majority variations are on the residues with high conservation. 25 variations are predicted to be pathogenic by PON-P2 and they are all in high conservation positions. Some variations in very low conservation sites, like p.Gln831Arg, p.Ala659Val and p.Phe167Leu, are all considered to be neutral by PON-P2. There is no stability prediction results since the structure of these two proteins are not available in PDB.

In total 101 variations from 10 different proteins are found for DMD, the analysis result is shown in Supplementary Table S4. Most residues are high conserved predicted by SIFT. Three proteins, solute carrier family 2 and facilitated glucose transporter member 1 (gene: SLC2A1), interferon-inducible double-stranded RNA-dependent protein kinase activator A isoform 1 (gene: PRKRA) and serine-protein kinase ATM isoform a (gene: ATM) have their structures reported in PDB, all the 19 variations found on them are predicted to decrease the protein stability by our method. Variations of 61 are predicted to be pathogenic by PON-P2 and all of them are in high conservation positions with extremely low SIFT scores.

In total 142 variations from 20 different proteins related to FTLD are analysed (Supplementary Table S5). Some variations are in low conservation position predicted by SIFT and most of these variations are considered not pathogenic by PON-P2 (unknown or neutral). Variations from 15 proteins can be analysed by PPSC since the structures are available. Only two variations, p.Lys238Glu in protein sequestosome-1 isoform 1 (gene: SQSTM1) and p.Lys263Glu in protein TAR DNA-binding protein 43 (gene: TARDBP) can increase protein stability using PPSC prediction while others are all predicted to decrease protein stability. Usually the variations that are considered as pathogenic by PON-P2 are in high conservation positions. But for FTLD related cases, there are a few exceptions: e.g. p.Leu424Val in protein presenilin-1 isoform I-467 (gene: PSEN1), p.Pro348Leu in protein sequestosome-1 isoform 1 (gene: SQSTM1) and 8 variation in protein transitional endoplasmic reticulum ATPase (VCP).

Variations found in more than one disease were also collected and analysed. There are 67 such variations found in 25 proteins (Supplementary Table S6). Majority of variations are happened at the conserved sites, only a few exceptions, e.g. p.Ile723Val, p.Val380Leu and p.Ala53Thr are predicted very low conserved (Supplementary Table S7). Of 39 variations with structure information, 33 are predicted decreasing the protein stability. About one third of the variations (24 out of 67) are predicted to be pathogenic by PON-P2. But there is no direct relationship found with conservation or stability prediction result. Since the dataset is not big enough, further studies are required in future.

### Future work and perspectives

The primary objective of this work is to design a disease-centric resource for further data analysis and clinical research. We will make the database open to data submission and expert checking, and try to develop data mining tools to collect and update data from existing database automatically. In addition, more tools will also be developed for the analyses and applications of the variations.

With the paradigm shifting toward personalized medicine and precision medicine, the personal phenotyping data will be collected for the precision mapping to the genotyping information (21, 22). The NDDVD database will be updated with more personalized and paired genotyping-phenotyping data for systems or network level modeling (23), which will be helpful to the future screening of high risk NDD population and personalized diagnosis and treatment of NDD patients.

## Supplementary data

Supplementary data are available at *Database* Online.

## Funding

This work is supported by the National Key Research and Development Program of China (No. 2016YFC1306605), the National Nature Science Foundation of China (Grant No. 31670851, 31470821, 91530320, 61602332, 31600671) and the University Science Research Project of Jiangsu Province (No.14KJB520035).

*Conflict of interest*. None declared.

## Supplementary Material

Supplementary Data
