# Supplementary material for: NDDVD: an integrated and manually curated Neurodegenerative Diseases Variation Database
Source: Database (Oxford). 2018 Mar 5;2018:bay018. doi: 10.1093/database/bay018 (PMC5841369; doi:10.1093/database/bay018)
Supplement: Supplementary Data [file bay018_supp.doc]

**Supplementary for**

**NDDVD: an integrated and manually curated Neurodegenerative Diseases Variation Database**

Yang Yang^1,2,3^, Chen Xu^1^, Xingyun Liu^1^, Chao Xu^1^, Yuanyuan Zhang^1^, Li Shen^1,4^, Mauno Vihinen^3^ and Bairong Shen^1, *^

^1^Center for Systems Biology, Soochow University, 215006, China

^2^School of Computer Science and Technology, Soochow University, 215006, China

^3^Department of Experimental Medical Science, Lund University, BMC B13, SE-221 84 Lund, Sweden

^4^Department of Genetics & Systems Biology Institute, Yale University School of Medicine, West Haven, CT 06516, USA

## Steps of manually variant-screening process


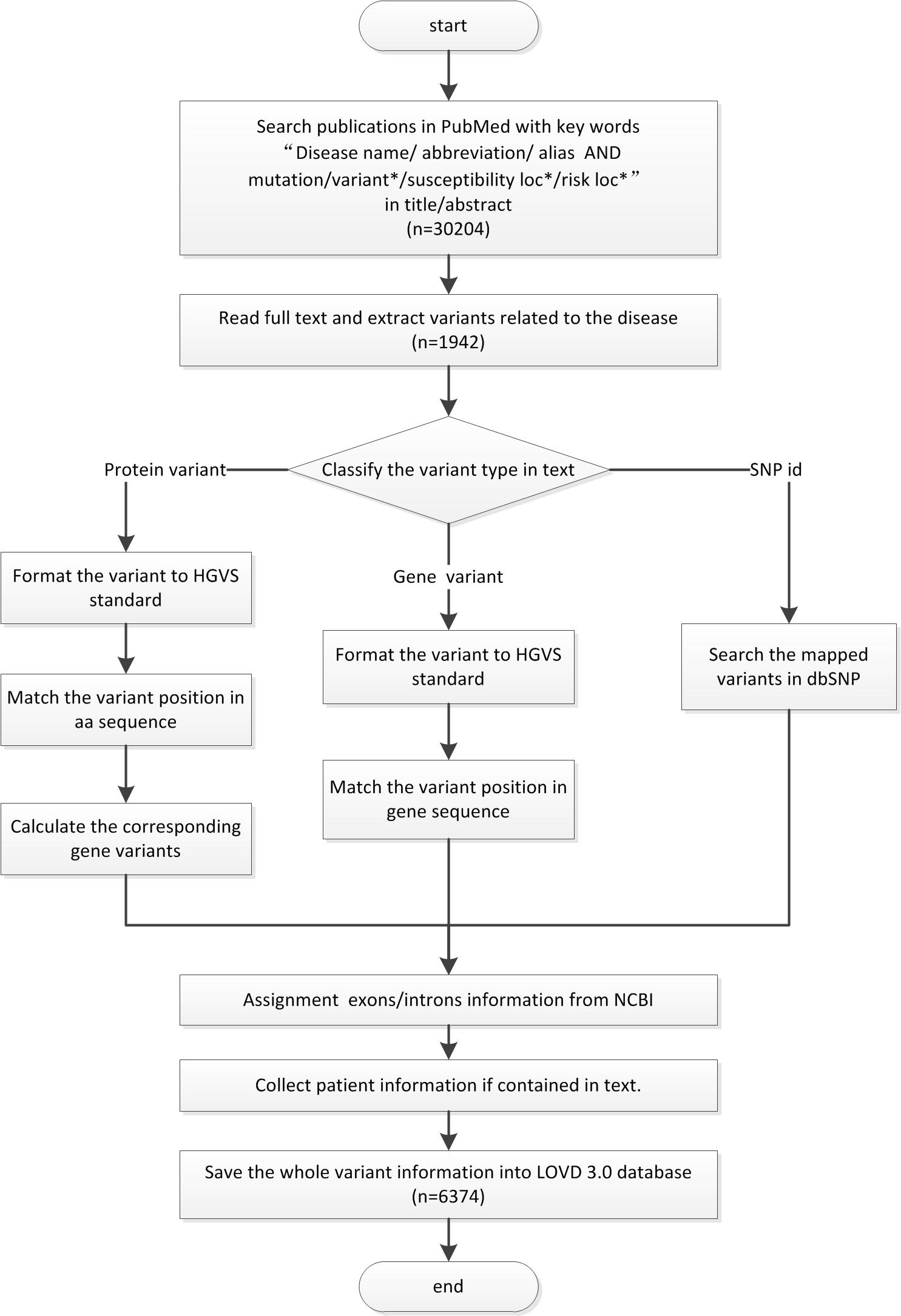


Supplementary figure 1 steps of manually variant-screening process

## Aliases of diseases searched

Supplementary Table 1 Aliases of diseases

| **No.** | **Disease^a^** | **Aliases** |
| --- | --- | --- |
| 1 | Alexander Disease | Alexander disease, fibrinoid leukodystrophy |
| 2 | Amyotrophic Lateral Sclerosis | Amyotrophic lateral sclerosis (ALS), motor neurone disease (MND), Lou Gehrig's disease |
| 3 | Alzheimer Disease | Alzheimer's disease, Alzheimer's |
| 4 | Canavan Disease | Canavan disease, Canavan–van Bogaert–Bertrand disease |
| 5 | Cockayne Syndrome | Cockayne syndrome, Cockayne's, Neill-Dingwall syndrome |
| 6 | [Creutzfeldt-Jakob Disease](http://127.0.0.1/new/diseasedetail.php?diseasename=Creutzfeldt-Jakob disease) | Creutzfeldt–Jakob disease (CJD) |
| 7 | Dystonia Musculorum Deformans | Dystonia musculorum deformans(DMD) |
| 8 | Familial Amyloid Neuropathy | familial amyloid neuropathies, familial amyloidotic neuropathies,  neuropathic heredofamilial amyloidosis, familial amyloid polyneuropathy |
| 9 | Fatal Familial Insomnia | Fatal familial insomnia (FFI) |
| 10 | Frontotemporal Lobar Degeneration | Frontotemporal lobar degeneration (FTLD), Frontotemporal dementia (FTD) |
| 11 | Gerstmann-Straussler-Scheinker Disease | Gerstmann–Sträussler–Scheinker syndrome (GSS) |
| 12 | Hepatolenticular Degeneration | Hepatolenticular Degeneration, Wilson's disease, Wilson disease |
| 13 | Hereditary sensory and Autonomic Neuropathy | Hereditary sensory and autonomic neuropathy (HSAN), hereditary sensory neuropathy (HSN) |
| 14 | Hereditary Sensory and Motor Neuropathy | Hereditary motor and sensory neuropathies (HMSN), Hereditary Sensory and Motor Neuropathy, Charcot–Marie–Tooth disease (CMT) |
| 15 | Lafora Disease | Lafora disease, Lafora progressive myoclonic epilepsy, MELF |
| 16 | Lesch-Nyhan Syndrome | Lesch–Nyhan syndrome (LNS), Nyhan syndrome, Lesch–Nyhan disease,  Nyhan disease, juvenile gout |
| 17 | Lewy Body Dementia | Lewy body dementia (LBD), Dementia with Lewy bodies (DLB), diffuse Lewy body disease, cortical Lewy body disease, senile dementia of Lewy type |
| 18 | Menkes Kinky Hair Syndrome | Menkes disease (MNK), Menkes syndrome, Trichopoliodystrophy, copper transport disease,  steely hair disease, kinky hair disease |
| 19 | Multiple System Atrophy | Multiple system atrophy (MSA), Shy-Drager syndrome |
| 20 | Myotonia Congenita | Congenital myotonia, myotonia congenita |
| 21 | Neuronal Ceroid-Lipofuscinoses | Neuronal ceroid lipofuscinosis (NCL), Neuronal Ceroid Lipofuscinoses |
| 22 | Optic Atrophy | Optic atrophy, Optic neuropathy |
| 23 | Pantothenate Kinase-Associated Neurodegeneration | Pantothenate kinase-associated neurodegeneration (PKAN), neurodegeneration with brain iron accumulation 1 (NBIA1), Hallervorden–Spatz syndrome |
| 24 | Parkinson Disease | Parkinson Disease, Parkinson's disease |
| 25 | Pelizaeus–Merzbacher Disease | Pelizaeus–Merzbacher disease (PMD) |
| 26 | Rett Syndrome | Rett syndrome (RTT), Cerebroatrophic hyperammonemia |
| 27 | Spinal Muscular Atrophies of Childhood | Spinal muscular atrophies of childhood, Spinal muscular atrophy (SMA) |
| 28 | Spinal Muscular Atrophy of Adults | Spinal muscular atrophy of Adults, Spinal muscular atrophy (SMA) |
| 29 | Spinocerebellar Degenerations | Spinocerebellar ataxia (SCA), spinocerebellar atrophy, spinocerebellar degeneration |
| 30 | Tuberous Sclerosis | Tuberous Sclerosis, Tuberous sclerosis complex (TSC) |
| 31 | Unverricht-Lundborg Syndrome | Unverricht-Lundborg Syndrome, Unverricht–Lundborg disease (ULD or EPM1) |

## Data statistics according to the patient’s country of origin in NDDMD

Supplementary Table 2 origin of the patients

| **No.** | **Disease ^a^** | **US** | **CHN^b^** | **GER^c^** | **ITA^d^** | **JPN^e^** | **SPN^f^** | **UK** | **Eur^g^** | **Others** | **total** |
| --- | --- | --- | --- | --- | --- | --- | --- | --- | --- | --- | --- |
| 1 | Alexander Disease | 2 | 2 | 2 | 3 | 14 | 1 |  |  | 3 | 27 |
| 2 | Amyotrophic Lateral Sclerosis | 7 | 45 | 5 | 8 | 14 | 3 | 35 | 4 | 27 | 148 |
| 3 | Alzheimer Disease | 2 | 39 |  | 5 | 33 | 2 | 1 | 5 | 25 | 112 |
| 4 | Canavan Disease |  |  |  |  | 1 |  | 1 | 7 | 11 | 20 |
| 5 | Cockayne Syndrome |  | 9 | 1 | 1 |  |  |  |  | 1 | 12 |
| 6 | [Creutzfeldt-Jakob Disease](http://127.0.0.1/new/diseasedetail.php?diseasename=Creutzfeldt-Jakob disease) |  | 5 |  |  |  |  |  |  |  | 5 |
| 7 | Dystonia Musculorum Deformans |  | 4 | 1 | 3 | 3 | 1 | 2 | 5 | 15 | 34 |
| 8 | Familial Amyloid Neuropathy | 6 | 3 | 6 | 13 | 17 | 2 | 3 | 9 | 19 | 78 |
| 9 | Fatal Familial Insomnia | 1 |  |  |  | 2 |  |  |  | 4 | 7 |
| 10 | Frontotemporal Lobar Degeneration | 4 | 2 | 2 | 5 | 2 |  | 29 | 4 | 9 | 57 |
| 11 | Gerstmann-Straussler-Scheinker Disease | 1 |  |  |  |  |  |  |  | 1 | 2 |
| 12 | Hepatolenticular Degeneration |  | 69 |  | 2 | 1 | 2 |  | 1 | 3 | 78 |
| 13 | Hereditary sensory and Autonomic Neuropathy | 2 |  |  |  | 3 |  | 1 | 1 | 5 | 12 |
| 14 | Hereditary Sensory and Motor Neuropathy |  | 2 | 4 | 1 | 7 | 1 | 1 |  | 12 | 28 |
| 15 | Lafora Disease |  |  |  | 1 | 1 |  |  |  | 1 | 3 |
| 16 | Lesch-Nyhan Syndrome |  |  |  | 1 | 9 |  | 1 |  | 4 | 15 |
| 17 | Lewy Body Dementia |  |  |  |  |  | 1 |  |  | 3 | 4 |
| 18 | Menkes Kinky Hair Syndrome |  | 5 |  |  | 1 |  |  |  | 5 | 11 |
| 19 | Multiple System Atrophy |  | 7 |  |  | 6 |  |  |  |  | 13 |
| 20 | Myotonia Congenita |  | 4 | 2 | 1 | 4 | 10 |  | 4 | 9 | 34 |
| 21 | Neuronal Ceroid-Lipofuscinoses |  | 1 | 1 | 7 |  | 3 | 17 | 48 | 15 | 92 |
| 22 | Optic Atrophy |  | 9 | 1 | 2 | 5 |  | 2 | 4 | 1 | 24 |
| 23 | Pantothenate Kinase-Associated Neurodegeneration |  | 6 |  |  |  |  |  | 1 | 9 | 16 |
| 24 | Parkinson Disease |  | 27 |  | 8 |  | 1 |  | 9 | 4 | 49 |
| 25 | Pelizaeus–Merzbacher Disease | 1 | 6 | 1 |  | 7 |  | 1 |  | 4 | 20 |
| 26 | Rett Syndrome |  | 8 |  | 2 | 5 |  | 3 |  | 17 | 35 |
| 27 | Spinal Muscular Atrophies of Childhood |  | 6 |  |  |  |  |  |  |  | 6 |
| 28 | Spinal Muscular Atrophy of Adults |  | 12 |  |  |  |  |  |  |  | 12 |
| 29 | Spinocerebellar Degenerations |  | 2 |  |  |  |  |  | 1 |  | 3 |
| 30 | Tuberous Sclerosis |  | 4 |  |  |  |  |  |  | 1 | 5 |
| 31 | Unverricht-Lundborg Syndrome |  |  |  |  | 1 |  |  |  |  | 1 |
|  | total | 26 | 277 | 26 | 63 | 136 | 27 | 97 | 103 | 208 | 963 |

^a^ origin information of the patients is not included in every reference, so the disease list contains 31 individual diseases; ^b^ Chinese; ^c^ German; ^d^ Italian; ^e^ Japanese; ^f^ Spanish; ^g^ European (not in special European country)

## Analysis of MC related variations

Supplementary Table 3 Analysis of MC related variations

| Gene name | Transcript | Variations | Conservation | | Stability | | Pathogenic | | |
| --- | --- | --- | --- | --- | --- | --- | --- | --- | --- |
|  |  |  | SIFT | | PPSC | | | PON-P2 | |
|  |  |  | result | score | PDB | score | | result | score |
| SCN4A | NM_000334.4 | p.Val445Met | DEL | 0.02 |  |  | | Unknown | 0.723 |
|  |  | p.Thr704Met | DEL | 0 |  |  | | Unknown | 0.681 |
|  |  | p.Val1293Ile | DEL | 0 |  |  | | Unknown | 0.319 |
|  |  | p.Gly1306Glu | DEL | 0 |  |  | | Neutral | 0.116 |
|  |  | p.Thr1313Met | DEL | 0.01 |  |  | | Unknown | 0.402 |
|  |  | p.Met1476Ile | TOL | 0.06 |  |  | | Unknown | 0.351 |
| CLCN1 | NM_000083.2 | p.Met128Val | TOL | 0.21 |  |  | | Unknown | 0.318 |
|  |  | p.Met128Ile | TOL | 0.06 |  |  | | Unknown | 0.330 |
|  |  | p.Ser132Cys | DEL | 0.03 |  |  | | Unknown | 0.665 |
|  |  | p.Tyr137Asp | DEL | 0.04 |  |  | | Unknown | 0.722 |
|  |  | p.Val165Gly | DEL | 0 |  |  | | Unknown | 0.429 |
|  |  | p.Phe167Leu | TOL | 0.56 |  |  | | Neutral | 0.211 |
|  |  | p.Ser189Cys | DEL | 0 |  |  | | Pathogenic | 0.968 |
|  |  | p.Ser189Phe | DEL | 0 |  |  | | Pathogenic | 0.967 |
|  |  | p.Gly190Ser | DEL | 0 |  |  | | Unknown | 0.655 |
|  |  | p.Glu193Lys | DEL | 0 |  |  | | Pathogenic | 0.734 |
|  |  | p.Leu198Pro | DEL | 0 |  |  | | Pathogenic | 0.980 |
|  |  | p.Ala218Thr | TOL | 0.17 |  |  | | Unknown | 0.276 |
|  |  | p.Gly230Glu | DEL | 0.01 |  |  | | Unknown | 0.636 |
|  |  | p.Gly230Val | DEL | 0 |  |  | | Unknown | 0.607 |
|  |  | p.Gly233Val | DEL | 0 |  |  | | Unknown | 0.676 |
|  |  | p.Val236Leu | DEL | 0 |  |  | | Unknown | 0.391 |
|  |  | p.Tyr261Cys | DEL | 0.05 |  |  | | Unknown | 0.666 |
|  |  | p.Thr268Met | DEL | 0 |  |  | | Unknown | 0.270 |
|  |  | p.Leu283Phe | TOL | 0.09 |  |  | | Pathogenic | 0.974 |
|  |  | p.Gly285Glu | DEL | 0 |  |  | | Pathogenic | 0.974 |
|  |  | p.Val286Ala | DEL | 0.02 |  |  | | Pathogenic | 0.981 |
|  |  | p.Leu287Ile | DEL | 0 |  |  | | Pathogenic | 0.971 |
|  |  | p.Ile290Met | DEL | 0 |  |  | | Pathogenic | 0.980 |
|  |  | p.Glu291Lys | DEL | 0 |  |  | | Pathogenic | 0.764 |
|  |  | p.Ala298Thr | DEL | 0.01 |  |  | | Unknown | 0.683 |
|  |  | p.Tyr302His | DEL | 0 |  |  | | Unknown | 0.725 |
|  |  | p.Phe307Ser | DEL | 0 |  |  | | Unknown | 0.446 |
|  |  | p.Thr310Met | TOL | 0.18 |  |  | | Pathogenic | 0.713 |
|  |  | p.Ala313Thr | TOL | 0.13 |  |  | | Unknown | 0.704 |
|  |  | p.Arg317Gln | DEL | 0.03 |  |  | | Unknown | 0.690 |
|  |  | p.Ile329Thr | DEL | 0 |  |  | | Unknown | 0.627 |
|  |  | p.Ala331Thr | DEL | 0.03 |  |  | | Unknown | 0.675 |
|  |  | p.Arg338Gln | TOL | 0.26 |  |  | | Unknown | 0.693 |
|  |  | p.Met373Leu | TOL | 0.14 |  |  | | Unknown | 0.289 |
|  |  | p.Gln412Pro | DEL | 0.02 |  |  | | Unknown | 0.558 |
|  |  | p.Phe413Cys | DEL | 0 |  |  | | Unknown | 0.611 |
|  |  | p.Gly416Glu | DEL | 0 |  |  | | Pathogenic | 0.730 |
|  |  | p.Arg421Cys | DEL | 0.01 |  |  | | Unknown | 0.682 |
|  |  | p.Ile424Met | TOL | 0.26 |  |  | | Unknown | 0.441 |
|  |  | p.Trp433Arg | DEL | 0 |  |  | | Unknown | 0.658 |
|  |  | p.Ser471Phe | DEL | 0.02 |  |  | | Unknown | 0.551 |
|  |  | p.Pro480Thr | DEL | 0 |  |  | | Unknown | 0.723 |
|  |  | p.Pro480Ser | DEL | 0 |  |  | | Unknown | 0.715 |
|  |  | p.Pro480Leu | DEL | 0 |  |  | | Pathogenic | 0.733 |
|  |  | p.Gly482Arg | DEL | 0 |  |  | | Pathogenic | 0.748 |
|  |  | p.Phe484Leu | DEL | 0.03 |  |  | | Pathogenic | 0.960 |
|  |  | p.Met485Val | TOL | 0.6 |  |  | | Unknown | 0.620 |
|  |  | p.Arg496Ser | DEL | 0 |  |  | | Unknown | 0.615 |
|  |  | p.Gly499Arg | DEL | 0 |  |  | | Pathogenic | 0.756 |
|  |  | p.Gly523Asp | TOL | 0.06 |  |  | | Pathogenic | 0.765 |
|  |  | p.Tyr524Cys | DEL | 0 |  |  | | Pathogenic | 0.755 |
|  |  | p.Ala531Val | DEL | 0 |  |  | | Pathogenic | 0.984 |
|  |  | p.Val536Leu | DEL | 0 |  |  | | Pathogenic | 0.980 |
|  |  | p.Thr539Pro | DEL | 0 |  |  | | Pathogenic | 0.972 |
|  |  | p.Thr550Met | DEL | 0 |  |  | | Pathogenic | 0.984 |
|  |  | p.Gln552Arg | DEL | 0 |  |  | | Pathogenic | 0.981 |
|  |  | p.Ile553Phe | DEL | 0.02 |  |  | | Pathogenic | 0.978 |
|  |  | p.His555Asn | DEL | 0 |  |  | | Pathogenic | 0.982 |
|  |  | p.Ile556Asn | DEL | 0 |  |  | | Unknown | 0.646 |
|  |  | p.Met560Thr | DEL | 0 |  |  | | Unknown | 0.461 |
|  |  | p.Val563Ile | DEL | 0.01 |  |  | | Unknown | 0.478 |
|  |  | p.Asn567Lys | TOL | 0.32 |  |  | | Unknown | 0.642 |
|  |  | p.Pro575Ser | DEL | 0.03 |  |  | | Unknown | 0.715 |
|  |  | p.Met609Lys | DEL | 0 |  |  | | Unknown | 0.657 |
|  |  | p.Leu628Pro | DEL | 0.01 |  |  | | Unknown | 0.620 |
|  |  | p.Leu629Pro | DEL | 0 |  |  | | Unknown | 0.643 |
|  |  | p.Thr631Ile | TOL | 0.12 |  |  | | Unknown | 0.268 |
|  |  | p.Val640Gly | DEL | 0 |  |  | | Unknown | 0.400 |
|  |  | p.Asp644Gly | DEL | 0 |  |  | | Unknown | 0.315 |
|  |  | p.Ala659Val | TOL | 0.58 |  |  | | Neutral | 0.137 |
|  |  | p.Arg669Cys | DEL | 0 |  |  | | Unknown | 0.707 |
|  |  | p.Phe708Leu | TOL | 0.71 |  |  | | Neutral | 0.110 |
|  |  | p.Thr736Ile | TOL | 0.4 |  |  | | Neutral | 0.062 |
|  |  | p.Gln788Pro | TOL | 0.07 |  |  | | Unknown | 0.373 |
|  |  | p.Gln831Arg | TOL | 1 |  |  | | Neutral | 0.093 |
|  |  | p.Leu844Phe | DEL | 0 |  |  | | Neutral | 0.143 |
|  |  | p.Pro932Leu | TOL | 0.26 |  |  | | Neutral | 0.112 |

## Analysis of DMD related variations

Supplementary Table 4 Analysis of DMD related varaitions

| Gene name | Transcript | Variations | Conservation | | | Stability | | | Pathogenic | |
| --- | --- | --- | --- | --- | --- | --- | --- | --- | --- | --- |
|  |  |  | SIFT | | PPSC | | | PON-P2 | | |
|  |  |  | result | score | PDB | | score | result | | score |
| SLC2A1 | NM_006516.2 | p.Arg468Trp | DEL | 0 | 1SUK | | -1.117 | Pathogenic | | 0.897 |
|  |  | p.Arg333Trp | DEL | 0 | 1SUK | | -1.117 | Pathogenic | | 0.915 |
|  |  | p.Gly314Ser | DEL | 0 | 1SUK | | -1.121 | Pathogenic | | 0.812 |
|  |  | p.Ala275Thr | DEL | 0 | 1SUK | | -1.123 | Unknown | | 0.712 |
|  |  | p.Lys256Trp | DEL | 0 | 1SUK | | -1.126 | Pathogenic | | 0.778 |
|  |  | p.Arg126Leu | DEL | 0 | 1SUK | | -1.117 | Pathogenic | | 0.811 |
|  |  | p.Arg126His | DEL | 0 | 1SUK | | -1.12 | Unknown | | 0.680 |
|  |  | p.Arg126Cys | DEL | 0 | 1SUK | | -1.121 | Pathogenic | | 0.892 |
|  |  | p.Ser95Ile | DEL | 0.01 | 1SUK | | -1.123 | Pathogenic | | 0.853 |
|  |  | p.Arg93Trp | DEL | 0.01 | 1SUK | | -1.123 | Pathogenic | | 0.915 |
|  |  | p.Gly91Asp | DEL | 0 | 1SUK | | -1.132 | Pathogenic | | 0.936 |
|  |  | p.Asn34Ile | DEL | 0 | 1SUK | | -1.114 | Pathogenic | | 0.755 |
| ATP1A3 | NM_152296.4 | p.Asp923Asn | DEL | 0.05 |  | |  | Pathogenic | | 0.793 |
|  |  | p.Gly867Asp | DEL | 0 |  | |  | Pathogenic | | 0.899 |
|  |  | p.Asp801Tyr | DEL | 0 |  | |  | Pathogenic | | 0.854 |
|  |  | p.Phe780Leu | DEL | 0 |  | |  | Unknown | | 0.788 |
|  |  | p.Ile758Ser | DEL | 0 |  | |  | Unknown | | 0.842 |
|  |  | p.Thr613Met | DEL | 0 |  | |  | Unknown | | 0.801 |
|  |  | p.Glu277Lys | DEL | 0 |  | |  | Pathogenic | | 0.815 |
|  |  | p.Ile274Thr | DEL | 0 |  | |  | Unknown | | 0.852 |
| TOR1A | NM_000113.2 | p.Arg288Gln | TOL | 0.23 |  | |  | Pathogenic | | 0.856 |
|  |  | p.Asp216His | DEL | 0.02 |  | |  | Unknown | | 0.330 |
|  |  | p.Phe205Ile | DEL | 0 |  | |  | Pathogenic | | 0.934 |
|  |  | p.Asp194Val | DEL | 0.02 |  | |  | Unknown | | 0.834 |
|  |  | p.Val129Ile | TOL | 0.09 |  | |  | Unknown | | 0.675 |
|  |  | p.Glu121Lys | TOL | 1 |  | |  | Unknown | | 0.287 |
| GCH1 | NM_000161.2 | p.Met1Leu | TOL | 0.06 |  | |  | Unknown | | 0.286 |
|  |  | p.Ser19Cys | DEL | 0.05 |  | |  | Unknown | | 0.321 |
|  |  | p.Pro23Leu | TOL | 0.44 |  | |  | Unknown | | 0.555 |
|  |  | p.Leu71Gln | DEL | 0 |  | |  | Pathogenic | | 0.907 |
|  |  | p.Ala74Val | DEL | 0.01 |  | |  | Pathogenic | | 0.890 |
|  |  | p.Tyr75Cys | DEL | 0.02 |  | |  | Pathogenic | | 0.903 |
|  |  | p.Leu79Pro | DEL | 0 |  | |  | Pathogenic | | 0.990 |
|  |  | p.Gly83Ala | DEL | 0.01 |  | |  | Pathogenic | | 0.938 |
|  |  | p.Arg88Trp | DEL | 0 |  | |  | Pathogenic | | 0.984 |
|  |  | p.Arg88Pro | DEL | 0 |  | |  | Pathogenic | | 0.949 |
|  |  | p.Gly90Val | DEL | 0 |  | |  | Pathogenic | | 0.981 |
|  |  | p.Thr94Met | DEL | 0 |  | |  | Pathogenic | | 0.957 |
|  |  | p.Ala98Val | TOL | 1 |  | |  | Pathogenic | | 0.852 |
|  |  | p.Met102Lys | DEL | 0 |  | |  | Pathogenic | | 0.959 |
|  |  | p.Met102Arg | DEL | 0 |  | |  | Pathogenic | | 0.959 |
|  |  | p.Thr106Ile | DEL | 0 |  | |  | Pathogenic | | 0.898 |
|  |  | p.Gly108Asp | DEL | 0 |  | |  | Pathogenic | | 0.985 |
|  |  | p.Asp115Asn | DEL | 0.03 |  | |  | Unknown | | 0.721 |
|  |  | p.Asp134Val | DEL | 0 |  | |  | Pathogenic | | 0.983 |
|  |  | p.Ile135Lys | DEL | 0 |  | |  | Pathogenic | | 0.967 |
|  |  | p.Ile135Thr | DEL | 0 |  | |  | Pathogenic | | 0.960 |
|  |  | p.Met137Arg | DEL | 0 |  | |  | Pathogenic | | 0.908 |
|  |  | p.Cys141Arg | DEL | 0 |  | |  | Pathogenic | | 0.976 |
|  |  | p.Cys141Trp | DEL | 0 |  | |  | Pathogenic | | 0.891 |
|  |  | p.His144Pro | DEL | 0 |  | |  | Pathogenic | | 0.927 |
|  |  | p.Leu145Phe | DEL | 0 |  | |  | Unknown | | 0.756 |
|  |  | p.His153Pro | DEL | 0 |  | |  | Pathogenic | | 0.878 |
|  |  | p.Gly155Ser | DEL | 0 |  | |  | Pathogenic | | 0.824 |
|  |  | p.Leu163Arg | DEL | 0 |  | |  | Pathogenic | | 0.962 |
|  |  | p.Ser176Thr | DEL | 0.01 |  | |  | Unknown | | 0.796 |
|  |  | p.Arg178Ser | DEL | 0 |  | |  | Pathogenic | | 0.923 |
|  |  | p.Gln180Arg | DEL | 0 |  | |  | Pathogenic | | 0.939 |
|  |  | p.Thr186Lys | DEL | 0 |  | |  | Pathogenic | | 0.973 |
|  |  | p.Val191Ile | TOL | 0.41 |  | |  | Unknown | | 0.586 |
|  |  | p.Pro199Leu | DEL | 0 |  | |  | Pathogenic | | 0.967 |
|  |  | p.Gly201Glu | DEL | 0 |  | |  | Pathogenic | | 0.978 |
|  |  | p.Gly203Arg | DEL | 0 |  | |  | Pathogenic | | 0.935 |
|  |  | p.Val204Ile | DEL | 0 |  | |  | Pathogenic | | 0.838 |
|  |  | p.Ala208Glu | DEL | 0 |  | |  | Pathogenic | | 0.908 |
|  |  | p.Met211Val | DEL | 0 |  | |  | Pathogenic | | 0.989 |
|  |  | p.Met213Val | DEL | 0.02 |  | |  | Pathogenic | | 0.989 |
|  |  | p.Met221Thr | TOL | 0.59 |  | |  | Unknown | | 0.870 |
|  |  | p.Lys224Arg | TOL | 0.48 |  | |  | Unknown | | 0.639 |
|  |  | p.Met230Ile | DEL | 0 |  | |  | Pathogenic | | 0.988 |
|  |  | p.Gly232Val | DEL | 0 |  | |  | Pathogenic | | 0.956 |
|  |  | p.Phe234Ser | DEL | 0 |  | |  | Pathogenic | | 0.963 |
|  |  | p.Arg241Trp | DEL | 0 |  | |  | Pathogenic | | 0.988 |
|  |  | p.Arg249Ser | DEL | 0.07 |  | |  | Unknown | | 0.724 |
| PRKRA | NM_003690.4 | p.Pro222Leu | TOL | 0.07 | 2DIX | | -0.82 | Unknown | | 0.472 |
|  |  | p.Thr34Ser | TOL | 0.34 | 2DIX | | -0.33 | Unknown | | 0.307 |
|  |  | p.Asn102Ser | TOL | 0.56 | 2DIX | | -0.56 | Unknown | | 0.288 |
| SGCE | NM_001099401.1 | p.Leu196Arg | DEL | 0 |  | |  | Pathogenic | | 0.777 |
|  |  | p.Ile129Thr | DEL | 0 |  | |  | Unknown | | 0.656 |
|  |  | p.Arg102Gln | DEL | 0.01 |  | |  | Pathogenic | | 0.806 |
| PNKD | NM_015488.4 | p.Ala9Val | DEL | 0 |  | |  | Neutral | | 0.141 |
|  |  | p.Ala7Val | DEL | 0 |  | |  | Neutral | | 0.229 |
|  |  | p.Ala33Pro | DEL | 0.01 |  | |  | Neutral | | 0.087 |
| THAP1 | NM_018105.2 | p.Leu180Ser | DEL | 0.01 |  | |  | Pathogenic | | 0.947 |
|  |  | p.Glu174Gly | TOL | 0.19 |  | |  | Pathogenic | | 0.859 |
|  |  | p.His150Pro | TOL | 0.31 |  | |  | Unknown | | 0.720 |
|  |  | p.Lys89Arg | TOL | 0.26 |  | |  | Unknown | | 0.246 |
|  |  | p.Phe81Leu | DEL | 0.03 |  | |  | Unknown | | 0.658 |
|  |  | p.Ile80Val | TOL | 0.41 |  | |  | Unknown | | 0.407 |
|  |  | p.Asn75Ile | DEL | 0.01 |  | |  | Unknown | | 0.608 |
|  |  | p.Glu56Gly | TOL | 0.2 |  | |  | Unknown | | 0.378 |
|  |  | p.Leu32His | TOL | 0.15 |  | |  | Pathogenic | | 0.892 |
|  |  | p.Arg13His | DEL | 0.02 |  | |  | Unknown | | 0.748 |
|  |  | p.Gly9Cys | TOL | 0.14 |  | |  | Unknown | | 0.740 |
|  |  | p.Ser6Phe | TOL | 0.11 |  | |  | Unknown | | 0.539 |
|  |  | p.Cys5Trp | DEL | 0 |  | |  | Unknown | | 0.543 |
| ATM | NM_000051.3 | p.Ile191Asn | DEL | 0 | 5NP0 | | -0.848 | Pathogenic | | 0.914 |
|  |  | p.Glu2052Lys | DEL | 0.02 | 5NP0 | | -0.841 | Pathogenic | | 0.820 |
|  |  | p.Ala2067Asp | DEL | 0.01 | 5NP0 | | -0.885 | Unknown | | 0.774 |
|  |  | p.Arg2227Cys | DEL | 0 | 5NP0 | | -0.850 | Pathogenic | | 0.970 |
| GNAL | NM_001142339.2 | p.Phe133Leu | TOL | 1 |  | |  | Unknown | | 0.340 |

## Analysis of FTLD related variations

Supplementary Table 5 Analysis of FTLD related variations

| Gene name | Transcript | Variations | Conservation | | | Stability | | Pathogenic | |
| --- | --- | --- | --- | --- | --- | --- | --- | --- | --- |
|  |  |  | SIFT | | PPSC | | | PON-P2 | |
|  |  |  | result | score | PDB | | score | result | score |
| C9orf72 | NM_001256054.2 | p.Asn207Ser | TOL | 0.59 |  | |  | unknown | 0.276 |
| CCNF | NM_001761.2 | p.Ser3Gly | DEL | 0.02 |  | |  | Unknown | 0.248 |
|  |  | p.Arg392Thr | DEL | 0.02 |  | |  | Unknown | 0.670 |
| CHCHD10 | NM_213720.1 | p.His22Tyr | TOL | 0.32 |  | |  | Unkonwn | 0.229 |
|  |  | p.Pro23Ser | TOL | 0.12 |  | |  | Neutral | 0.156 |
|  |  | p.Pro23Leu | TOL | 0.08 |  | |  | Neutral | 0.169 |
|  |  | p.Ala32Asp | TOL | 0.16 |  | |  | Unkonwn | 0.486 |
|  |  | p.Pro34Ser | TOL | 0.38 |  | |  | Neutral | 0.182 |
|  |  | p.Val57Glu | DEL | 0.03 |  | |  | Pathogenic | 0.851 |
| CHMP2B | NM_014043.3 | p.Ile29Val | DEL | 0.01 | 2JQK | | NA | Neutral | 0.081 |
|  |  | p.Ser187Asn | TOL | 0.21 |  | | -0.491 | Neutral | 0.053 |
|  |  | p.Ser194Leu | TOL | 0.2 |  | | -0.571 | Unknown | 0.690 |
| FUS | NM_004960.3 | p.Gln140His | DEL | 0.03 | 4FQ3 | | NA | Unknown | 0.250 |
|  |  | p.Met254Val | TOL | 0.55 |  | | NA | Neutral | 0.228 |
|  |  | p.Arg521His | TOL | 0.21 |  | | NA | Unknown | 0.502 |
| GFAP | NM_002055.4 | p.Asp295Asn | NA | NA |  | |  | Pathogenic | 0.869 |
| GRN | NM_002087.2 | p.Met1Thr | DEL | 0 | 2JYE | | -1.049 | Unknown | 0.744 |
|  |  | p.Met1Ile | DEL | 0 |  | | -1.199 | Unknown | 0.471 |
|  |  | p.Ala9Asp | DEL | 0.04 |  | | -0.654 | Unknown | 0.686 |
|  |  | p.Cys105Tyr | DEL | 0 |  | | -0.689 | Pathogenic | 0.855 |
|  |  | p.Ser120Tyr | DEL | 0.04 |  | | -0.771 | Neutral | 0.241 |
|  |  | p.Cys139Arg | DEL | 0 |  | | -0.493 | Pathogenic | 0.876 |
|  |  | p.Thr182Met | TOL | 0.11 |  | | -0.408 | Neutral | 0.074 |
|  |  | p.Ala199Val | TOL | 0.53 |  | | -0.591 | Neutral | 0.094 |
|  |  | p.Pro248Leu | DEL | 0 |  | | -0.758 | Unknown | 0.742 |
|  |  | p.Arg298His | TOL | 0.13 |  | | -0.383 | Unknown | 0.603 |
|  |  | p.Ala324Thr | TOL | 0.56 |  | | -0.800 | Neutral | 0.056 |
|  |  | p.Pro373Ser | DEL | 0.02 |  | | -1.123 | Unknown | 0.456 |
|  |  | p.Thr409Met | TOL | 0.23 |  | | -0.408 | Neutral | 0.059 |
|  |  | p.Arg432Cys | TOL | 0.19 |  | | -0.544 | Neutral | 0.063 |
|  |  | p.Arg433Trp | DEL | 0.02 |  | | -0.260 | Unknown | 0.423 |
|  |  | p.Leu469Phe | TOL | 0.14 |  | | -1.142 | Neutral | 0.127 |
|  |  | p.Trp541Cys | DEL | 0.02 |  | | -0.842 | Pathogenic | 0.859 |
| LRRK2 | NM_198578.3 | p.Gly2019Ser | DEL | 0 | 4YZM | | -1.307 | Pathogenic | 0.893 |
| MAPT | NM_005910.5 | p.Arg5His | TOL | 0.14 | 5O3L | | -1.123 | Unknown | 0.428 |
|  |  | p.Asp13Gly | DEL | 0 |  | | -1.118 | Neutral | 0.078 |
|  |  | p.Gly55Arg | TOL | 0.37 |  | | -1.126 | Unknown | 0.317 |
|  |  | p.Val75Ala | TOL | 0.76 |  | | -1.117 | Neutral | 0.200 |
|  |  | p.Ala152Thr | TOL | 0.28 |  | | -1.123 | Unknown | 0.357 |
|  |  | p.Gln230Arg | TOL | 1 |  | | -1.116 | Unknown | 0.254 |
|  |  | p.Ala239Thr | TOL | 0.39 |  | | -1.123 | Neutral | 0.234 |
|  |  | p.Lys257Thr | DEL | 0 |  | | -1.129 | Unknown | 0.339 |
|  |  | p.Ile260Val | TOL | 0.27 |  | | -1.118 | Unknown | 0.469 |
|  |  | p.Leu266Val | DEL | 0.05 |  | | -1.122 | Unknown | 0.773 |
|  |  | p.Gly272Val | DEL | 0 |  | | -1.123 | Pathogenic | 0.986 |
|  |  | p.Gly273Arg | DEL | 0 |  | | -1.126 | Pathogenic | 0.971 |
|  |  | p.Asn279Lys | DEL | 0.01 |  | | -1.104 | Unknown | 0.536 |
|  |  | p.Leu284Arg | DEL | 0.03 |  | | -1.125 | Pathogenic | 0.906 |
|  |  | p.Asn296Asp | DEL | 0 |  | | -1.111 | Unknown | 0.779 |
|  |  | p.Asn296His | DEL | 0 |  | | -1.120 | Pathogenic | 0.868 |
|  |  | p.Pro301Leu | DEL | 0 |  | | -1.117 | Pathogenic | 0.907 |
|  |  | p.Pro301Ser | DEL | 0 |  | | -1.123 | Pathogenic | 0.862 |
|  |  | p.Pro301Thr | DEL | 0 |  | | -1.124 | Pathogenic | 0.855 |
|  |  | p.Ser305Ile | DEL | 0.01 |  | | -1.122 | Unknown | 0.593 |
|  |  | p.Ser305Asn | TOL | 1 |  | | -1.122 | Unknown | 0.549 |
|  |  | p.Leu315Arg | DEL | 0 |  | | -1.126 | Pathogenic | 0.952 |
|  |  | p.Lys317Met | DEL | 0 |  | | -1.118 | Unknown | 0.711 |
|  |  | p.Ser320Phe | DEL | 0 |  | | -1.120 | Unknown | 0.809 |
|  |  | p.Pro332Ser | DEL | 0 |  | | -1.121 | Pathogenic | 0.832 |
|  |  | p.Gly335Ser | DEL | 0 |  | | -1.130 | Pathogenic | 0.774 |
|  |  | p.Gly335Val | DEL | 0 |  | | -1.126 | Pathogenic | 0.918 |
|  |  | p.Gln336His | TOL | 0.21 |  | | -1.118 | Unknown | 0.441 |
|  |  | p.Gln336Arg | TOL | 0.53 |  | | -1.116 | Unknown | 0.402 |
|  |  | p.Val337Met | DEL | 0 |  | | -1.109 | Unknown | 0.837 |
|  |  | p.Glu342Val | TOL | 0.33 |  | | -1.115 | Unknown | 0.807 |
|  |  | p.Asp348Gly | DEL | 0 |  | | -1.117 | Unknown | 0.4 |
|  |  | p.Gln351Arg | DEL | 0.01 |  | | -1.118 | Pathogenic | 0.788 |
|  |  | p.Ser356Thr | DEL | 0 |  | | -1.122 | Unknown | 0.664 |
|  |  | p.Val363Ile | TOL | 0.14 |  | | -1.118 | Neutral | 0.212 |
|  |  | p.Pro364Ser | DEL | 0.02 |  | | -1.125 | Unknown | 0.360 |
|  |  | p.Gly366Arg | DEL | 0 |  | | -1.139 | Unknown | 0.708 |
|  |  | p.Lys369Ile | TOL | 0.7 |  | | -1.125 | Unknown | 0.452 |
|  |  | p.Gly389Arg | DEL | 0 |  | | -1.126 | Pathogenic | 0.937 |
|  |  | p.Arg406Trp | DEL | 0 |  | | -1.117 | Unknown | 0.581 |
|  |  | p.Thr427Met | DEL | 0 |  | | -1.113 | Pathogenic | 0.807 |
|  | NM_001123066.3 | p.Pro636Leu | DEL | 0.01 |  | | -1.123 | Pathogenic | 0.907 |
| OPTN | NM_021980.4 | p.Ala481Val | DEL | 0.05 | 2LUE | | -0.481 | Pathogenic | 0.787 |
| PRNP | NM_000311.3 | p.Pro39Leu | DEL | 0.01 | 1E1G | | -1.119 | Unknown | 0.539 |
| PSEN1 | NM_000021.3 | p.Leu113Pro | DEL | 0 | 2KR6 | | -1.339 | Pathogenic | 0.896 |
|  |  | p.Gly183Val | TOL | 0.23 |  | | -0.654 | Unknown | 0.809 |
|  |  | p.Leu226Phe | DEL | 0 |  | | -1.093 | Pathogenic | 0.932 |
|  |  | p.Met233Leu | TOL | 0.06 |  | | -0.604 | Pathogenic | 0.914 |
|  |  | p.Val412Ile | DEL | 0.04 |  | | -1.036 | Unknown | 0.855 |
|  |  | p.Leu424His | DEL | 0 |  | | -0.813 | Pathogenic | 0.918 |
|  |  | p.Leu424Val | TOL | 1 |  | | -0.698 | Pathogenic | 0.908 |
| SNCA | NM_000345.3 | p.Ala53Thr | TOL | 1 | 1XQ8 | | -1.127 | Unknown | 0.589 |
| SOD1 | NM_000454.4 | p.Ile113Thr | DEL | 0 |  | |  | Pathogenic | 0.861 |
| SQSTM1 | NM_003900.4 | p.Arg183Cys | DEL | 0.03 | 1Q02 | | -0.531 | Pathogenic | 0.900 |
|  |  | p.Arg212Cys | TOL | 0.09 |  | | -0.531 | Unknown | 0.437 |
|  |  | p.Lys238Glu | DEL | 0.04 |  | | 0.052 | Unknown | 0.703 |
|  |  | p.Glu274Asp | TOL | 0.61 |  | | -0.657 | Unknown | 0.285 |
|  |  | p.Glu319Lys | TOL | 0.91 |  | | -1.085 | Unknown | 0.362 |
|  |  | p.Asp329Gly | TOL | 0.61 |  | | -0.075 | Neutral | 0.240 |
|  |  | p.Leu341Val | TOL | 0.14 |  | | -0.783 | Unknown | 0.543 |
|  |  | p.Pro348Leu | TOL | 0.13 |  | | -0.770 | Pathogenic | 0.913 |
|  |  | p.Gly351Ala | DEL | 0.01 |  | | -0.911 | Unknown | 0.762 |
|  |  | p.Pro387Leu | DEL | 0.01 |  | | -0.770 | Pathogenic | 0.923 |
|  |  | p.Pro392Leu | DEL | 0 |  | | -0.770 | Pathogenic | 0.997 |
|  |  | p.Gly411Ser | DEL | 0 |  | | -0.935 | Pathogenic | 0.969 |
|  |  | p.Thr430Pro | DEL | 0.02 |  | | -1.189 | Pathogenic | 0.743 |
|  |  | p.Pro439Leu | TOL | 0.08 |  | | -0.770 | Unknown | 0.602 |
| TARDBP | NM_007375.3 | p.Lys263Glu | DEL | 0 | 2N3X | | 0.071 | Unknown | 0.546 |
|  |  | p.Asn267Ser | TOL | 0.9 |  | | -0.496 | Unknown | 0.491 |
|  |  | p.Gly295Ser | TOL | 0.58 |  | | -0.797 | Unknown | 0.576 |
|  |  | p.Met359Val | TOL | 0.51 |  | | -0.677 | Neutral | 0.196 |
|  |  | p.Arg361Thr | TOL | 0.35 |  | | -1.118 | Unknown | 0.427 |
|  |  | p.Ala382Thr | TOL | 0.39 |  | | -0.792 | Unknown | 0.588 |
|  |  | p.Ser393Leu | TOL | 0.09 |  | | -0.600 | Unknown | 0.714 |
| TBK1 | NM_013254.3 | p.Thr4Ala | DEL | 0.03 | 4IW0 | | -0.342 | Pathogenic | 0.930 |
|  |  | p.Leu306Ile | DEL | 0 |  | | -0.919 | Unknown | 0.682 |
|  |  | p.Arg384Thr | TOL | 0.65 |  | | -1.120 | Unknown | 0.634 |
|  |  | p.Lys401Glu | TOL | 0.14 |  | | -0.478 | Pathogenic | 0.928 |
|  |  | p.Met662Thr | TOL | 0.29 |  | | -0.551 | Unknown | 0.529 |
|  |  | p.Glu696Lys | TOL | 0.08 |  | | -1.057 | Unknown | 0.841 |
| TREM2 | NM_018965.3 | p.Ala28Val | TOL | 1 | 5ELI | | -1.120 | Unknown | 0.255 |
|  |  | p.Arg47His | TOL | 0.11 |  | | -1.120 | Unknown | 0.338 |
|  |  | p.Arg62His | TOL | 0.65 |  | | -1.119 | Neutral | 0.099 |
|  |  | p.Thr66Met | DEL | 0 |  | | -1.116 | Unknown | 0.320 |
|  |  | p.Thr96Lys | DEL | 0 |  | | -1.107 | Unknown | 0.387 |
|  |  | p.Ala105Thr | TOL | 0.07 |  | | -1.121 | Neutral | 0.045 |
|  |  | p.Leu211Pro | TOL | 0.3 |  | | -1.121 | Neutral | 0.097 |
| UBQLN2 | NM_013444.3 | p.Ala282Val | DEL | 0.02 |  | | -1.119 | Neutral | 0.044 |
|  |  | p.Pro497His | TOL | 0.19 | 2NBV | | -1.018 | Neutral | 0.191 |
| VCP | NM_007126.3 | p.Ile27Val | TOL | 0.82 | 3CF3 | | -1.117 | Unknown | 0.423 |
|  |  | p.Asn91Tyr | DEL | 0 |  | | -1.123 | Pathogenic | 0.961 |
|  |  | p.Arg93Cys | TOL | 0.06 |  | | -1.124 | Pathogenic | 0.950 |
|  |  | p.Arg95Gly | TOL | 0.66 |  | | -1.124 | Pathogenic | 0.893 |
|  |  | p.Gly97Glu | DEL | 0 |  | | -1.133 | Pathogenic | 0.963 |
|  |  | p.Ile126Phe | TOL | 0.22 |  | | -1.114 | Pathogenic | 0.912 |
|  |  | p.Pro137Leu | DEL | 0 |  | | -1.116 | Pathogenic | 0.966 |
|  |  | p.Arg155Cys | DEL | 0.01 |  | | -1.124 | Pathogenic | 0.994 |
|  |  | p.Arg155His | TOL | 0.06 |  | | -1.127 | Pathogenic | 0.976 |
|  |  | p.Arg155Leu | DEL | 0.05 |  | | -1.121 | Pathogenic | 0.962 |
|  |  | p.Gly157Arg | TOL | 0.33 |  | | -1.132 | Pathogenic | 0.977 |
|  |  | p.Arg159Cys | DEL | 0 |  | | -1.122 | Pathogenic | 0.992 |
|  |  | p.Arg191Gln | DEL | 0.03 |  | | -1.123 | Pathogenic | 0.988 |
|  |  | p.Leu198Trp | DEL | 0.01 |  | | -1.124 | Pathogenic | 0.917 |
|  |  | p.Ala232Glu | TOL | 0.23 |  | | -1.132 | Pathogenic | 0.977 |
|  |  | p.Thr262Ala | TOL | 0.23 |  | | -1.115 | Pathogenic | 0.949 |
|  |  | p.Asn387His | DEL | 0.01 |  | | -1.126 | Pathogenic | 0.960 |
|  |  | p.Ala439Pro | TOL | 0.32 |  | | -1.126 | Pathogenic | 0.815 |
|  |  | p.Lys524Ala | DEL | 0 |  | | -1.110 | Pathogenic | 0.778 |

## Analyzing Results for variations related to multiple diseases

Supplementary Table 6 variations related to more than one diseases

| Transcript | Variation | PDB | Gene | Protein | Diseases |
| --- | --- | --- | --- | --- | --- |
| NM_001761.2 | p.Ser3Gly |  | CCNF | cyclin-F isoform 1 | ALS, FTLD |
| NM_213720.1 | p.Pro34Ser |  | CHCHD10 | coiled-coil-helix-coiled-coil-helix domain-containing protein 10, mitochondrial isoform b precursor | ALS, FTLD |
| NM_014043.3 | p.Ile29Val |  | CHMP2B | charged multivesicular body protein 2b isoform 1 | ALS, FTLD |
| NM_015268.3 | p.Asn855Ser |  | DNAJC13 | dnaJ homolog subfamily C member 13 isoform 2 | DLB, PD |
| NM_004960.3 | p.Arg521His |  | FUS | RNA-binding protein FUS isoform 1 | ALS, FTLD |
| NM_002055.4 | p.Asp295Asn |  | GFAP | glial fibrillary acidic protein isoform 1 | ALXDRD, FTLD |
| NM_000520.4 | p.Val391Met |  | HEXA | beta-hexosaminidase subunit alpha preproprotein | SMAA, SMAC |
| NM_000410.3 | p.His63Asp | 1A6Z | HFE | hereditary hemochromatosis protein isoform 1 precursor | AD, ALS |
| NM_198578.3 | p.Asn551Lys |  | LRRK2 | leucine-rich repeat serine/threonine-protein kinase 2 | DLB, PD |
|  | p.Ile723Val |  |  |  | DLB, PD |
|  | p.Arg1398His |  |  |  | DLB, PD |
|  | p.Arg1514Gln |  |  |  | DLB, PD |
|  | p.Pro1542Ser |  |  |  | DLB, PD |
|  | p.Met1646Thr |  |  |  | DLB, PD |
|  | p.Ser1647Thr |  |  |  | DLB, PD |
|  | p.Gly2019Ser |  |  |  | DLB, FTLD, PD |
|  | p.Asn2081Asp |  |  |  | DLB, PD |
|  | p.Met2397Thr |  |  |  | DLB, PD |
| NM_005910.5 | p.Pro301Leu | 5O3L | MAPT | microtubule-associated protein tau isoform 2 | FTLD, PD, PSP |
| NM_004562.2 | p.Ser167Asn | 4BM9 | PARK2 | E3 ubiquitin-protein ligase parkin isoform 1 | PD, PSP |
|  | p.Cys212Tyr |  |  |  | PD, PSP |
|  | p.Val380Leu |  |  |  | PD, PSP |
|  | p.Asp394Asn |  |  |  | PD, PSP |
| NM_000311.3 | p.Pro105Leu | 1FKC | PRNP | major prion protein preproprotein | GSS, PD |
|  | p.Ala117Val |  |  |  | CJD, GSS |
|  | p.Met129Val |  |  |  | CJD, GSS, KURU |
|  | p.Val176Gly |  |  |  | CJD, GSS |
|  | p.Asp178Asn |  |  |  | AD, CJD, FFI, GSS |
|  | p.Glu196Lys |  |  |  | GSS, CJD |
|  | p.Glu200Lys |  |  |  | CJD, FFI |
|  | p.Ile215Val |  |  |  | AD, CJD |
|  | p.Tyr218Asn |  |  |  | CJD, GSS |
|  | p.Met232Arg |  |  |  | CJD, DLB |
| NM_000021.3 | p.Leu226Phe |  | PSEN1 | presenilin-1 isoform I-467 | AD, FTLD |
|  | p.Met233Leu |  |  |  | AD, FTLD |
| NM_000447.2 | p.Ala85Val |  | PSEN2 | presenilin-2 isoform 1 | AD, DLB |
| NM_004637.5 | p.Leu129Phe | 1YHN | RAB7A | ras-related protein Rab-7a | HMSN, HSAN |
|  | p.Lys157Asn |  |  |  | HMSN, HSAN |
|  | p.Asn161Thr |  |  |  | HMSN, HSAN |
|  | p.Val162Met |  |  |  | HMSN, HSAN |
| NM_000344.3 | p.Ala2Val | 1G5V | SMN1 | survival motor neuron protein isoform d | SMAA, SMAC |
|  | p.Ala111Gly |  |  |  | SMAA, SMAC |
|  | p.Tyr272Cys |  |  |  | SMAA, SMAC |
|  | p.Arg288Met | 4GLI |  |  | SMAA, SMAC |
| NM_000345.3 | p.Glu46Lys | 1XQ8 | SNCA | alpha-synuclein isoform NACP140 | DLB, PD |
|  | p.Ala53Thr |  |  |  | DLB, FTLD, PD |
| NM_000454.4 | p.Ile113Thr | 4FF9 | SOD1 | superoxide dismutase [Cu-Zn] | ALS, FTLD |
|  | p.Cys147Arg |  |  |  | ALS, PBP |
| NM_003900.4 | p.Glu274Asp |  | SQSTM1 | sequestosome-1 isoform 1 | AD, FTLD |
|  | p.Leu341Val |  |  |  | AD, FTLD |
|  | p.Pro392Leu |  |  |  | AD, FTLD |
| NM_007375.3 | p.Gly295Ser | 2N3X | TARDBP | TAR DNA-binding protein 43 | ALS, FTLD |
|  | p.Arg361Thr |  |  |  | ALS, FTLD |
|  | p.Ala382Thr |  |  |  | ALS, FTLD |
|  | p.Ser393Leu |  |  |  | ALS, FTLD |
| NM_013254.3 | p.Met662Thr |  | TBK1 | serine/threonine-protein kinase TBK1 | ALS, FTLD |
|  | p.Glu696Lys |  |  |  | ALS, FTLD |
| NM_018965.3 | p.Arg47His | 5ELI | TREM2 | triggering receptor expressed on myeloid cells 2 precursor isoform 1 precursor | AD, FTLD |
|  | p.Thr66Met |  |  |  | AD, FTLD |
| NM_000371.3 | p.Val50Met | 1F64 | TTR | transthyretin precursor | FAN, PBP |
|  | p.Ile127Val |  |  |  | FAN, PBP |
| NM_004738.4 | p.Pro56Ser | 2MDK | VAPB | vesicle-associated membrane protein-associated protein B/C isoform 1 | ALS, SMAA |
| NM_007126.3 | p.Asn91Tyr | 3QC8 | VCP | transitional endoplasmic reticulum ATPase | ALS, FTLD |
|  | p.Arg155Cys |  |  |  | ALS, FTLD |
|  | p.Arg155His |  |  |  | ALS, FTLD |
|  | p.Arg159Cys |  |  |  | ALS, FTLD |
|  | p.Ala232Glu |  |  |  | ALS, FTLD |

Supplementary Table 7 Analysis of variations related to multiple diseases

| Transcript | Variations | Conservation | | | Stability | | | Pathogenic | |
| --- | --- | --- | --- | --- | --- | --- | --- | --- | --- |
|  |  | SIFT | | PPSC | | | PON-P2 | | |
|  |  | result | score | PDB | | score | result | | score |
| NM_001761.2 | p.Ser3Gly | DEL | 0.02 |  | |  | Unknown | | 0.248 |
| NM_213720.1 | p.Pro34Ser | TOL | 0.38 |  | |  | Neutral | | 0.182 |
| NM_014043.3 | p.Ile29Val | DEL | 0.01 |  | |  | Neutral | | 0.081 |
| NM_015268.3 | p.Asn855Ser | TOL | 0.09 |  | |  | Neutral | | 0.163 |
| NM_004960.3 | p.Arg521His | TOL | 0.12 |  | |  | Unknown | | 0.502 |
| NM_002055.4 | p.Asp295Asn | NA | NA |  | |  | Pathogenic | | 0.869 |
| NM_000520.4 | p.Val391Met | DEL | 0.02 |  | |  | Unknown | | 0.595 |
| NM_000410.3 | p.His63Asp | DEL | 0 |  | |  | Pathogenic | | 0.846 |
| NM_198578.3 | p.Asn551Lys | TOL | 0.06 |  | |  | Pathogenic | | 0.821 |
|  | p.Ile723Val | TOL | 1 |  | |  | Unknown | | 0.496 |
|  | p.Arg1398His | TOL | 0.08 |  | |  | Unknown | | 0.539 |
|  | p.Arg1514Gln | TOL | 0.38 |  | |  | Unknown | | 0.597 |
|  | p.Pro1542Ser | TOL | 0.5 |  | |  | Pathogenic | | 0.831 |
|  | p.Met1646Thr | TOL | 0.37 |  | |  | Pathogenic | | 0.760 |
|  | p.Ser1647Thr | TOL | 0.62 |  | |  | Unknown | | 0.425 |
|  | p.Gly2019Ser | DEL | 0 |  | |  | Pathogenic | | 0.893 |
|  | p.Asn2081Asp | TOL | 0.07 |  | |  | Unknown | | 0.659 |
|  | p.Met2397Thr | TOL | 0.43 |  | |  | Unknown | | 0.464 |
| NM_016835.4 | p.Pro301Leu | DEL | 0 | 5O3L | | -1.117 | Pathogenic | | 0.907 |
| NM_004562.2 | p.Ser167Asn | TOL | 0.28 | 4BM9 | | -0.451 | Neutral | | 0.066 |
|  | p.Cys212Tyr | DEL | 0 | 4BM9 | | 0.005 | Unknown | | 0.766 |
|  | p.Val380Leu | TOL | 1 | 4BM9 | | -1.111 | Neutral | | 0.055 |
|  | p.Asp394Asn | DEL | 0.02 | 4BM9 | | 1.123 | Unknown | | 0.320 |
|  | p.Pro105Leu | DEL | 0 | 1E1G | | -0.756 | Unknown | | 0.795 |
| NM_000311.3 | p.Ala117Val | DEL | 0 | 1FKC | | -0.283 | Pathogenic | | 0.790 |
|  | p.Met129Val | DEL | 0.02 | 1E1G | | -0.737 | Unknown | | 0.498 |
|  | p.Val176Gly | DEL | 0 | 1E1G | | -3.165 | Unknown | | 0.550 |
|  | p.Asp178Asn | DEL | 0 | 1E1G | | -1.873 | Pathogenic | | 0.888 |
|  | p.Glu196Lys | DEL | 0 | 1E1G | | 0.241 | Unknown | | 0.741 |
|  | p.Glu200Lys | DEL | 0 | 1E1G | | -0.136 | Pathogenic | | 0.908 |
|  | p.Ile215Val | TOL | 0.18 | 1E1G | | -1.083 | Unknown | | 0.309 |
|  | p.Tyr218Asn | DEL | 0 | 1E1G | | -1.881 | Pathogenic | | 0.975 |
|  | p.Met232Arg | DEL | 0 | 1E1G | | 0.146 | Unknown | | 0.703 |
| NM_000021.3 | p.Leu226Phe | DEL | 0 |  | |  | Pathogenic | | 0.932 |
|  | p.Met233Leu | TOL | 0.06 |  | |  | Pathogenic | | 0.914 |
| NM_000447.2 | p.Ala85Val | DEL | 0.01 |  | |  | Pathogenic | | 0.863 |
| NM_004637.5 | p.Leu129Phe | DEL | 0.01 | 1YHN | | 0.132 | Unknown | | 0.83 |
|  | p.Lys157Asn | DEL | 0 | 1YHN | | -0.434 | Unknown | | 0.623 |
|  | p.Asn161Thr | DEL | 0 | 1YHN | | -0.904 | Unknown | | 0.706 |
|  | p.Val162Met | DEL | 0 | 1YHN | | -0.909 | Unknown | | 0.845 |
| NM_000344.3 | p.Ala2Val | DEL | 0.01 | 1G5V | | -0.382 | Neutral | | 0.098 |
|  | p.Ala111Gly | DEL | 0 | 1G5V | | -2.574 | Unknown | | 0.657 |
|  | p.Tyr272Cys | DEL | 0 | 1G5V | | 0.212 | Pathogenic | | 0.974 |
|  | p.Arg288Met | DEL | 0.01 | 4GLI | | -0.432 | Unknown | | 0.471 |
| NM_000345.3 | p.Glu46Lys | DEL | 0 | 1XQ8 | | -0.598 | Unknown | | 0.831 |
|  | p.Ala53Thr | TOL | 1 | 1XQ8 | | -0.369 | Unknown | | 0.589 |
| NM_000454.4 | p.Ile113Thr | DEL | 0 |  | |  | Pathogenic | | 0.861 |
|  | p.Cys147Arg | DEL | 0 |  | |  | Pathogenic | | 0.979 |
| NM_003900.4 | p.Glu274Asp | TOL | 0.61 |  | |  | Unknown | | 0.285 |
|  | p.Leu341Val | TOL | 0.14 |  | |  | Unknown | | 0.543 |
|  | p.Pro392Leu | DEL | 0 |  | |  | Pathogenic | | 0.997 |
| NM_007375.3 | p.Gly295Ser | TOL | 0.58 | 2N3X | | -1.123 | Unknown | | 0.576 |
|  | p.Arg361Thr | TOL | 0.35 | 2N3X | | -1.118 | Unknown | | 0.427 |
|  | p.Ala382Thr | TOL | 0.39 | 2N3X | | -1.123 | Unknown | | 0.588 |
|  | p.Ser393Leu | TOL | 0.09 | 2N3X | | -1.122 | Unknown | | 0.714 |
| NM_013254.3 | p.Met662Thr | TOL | 0.29 |  | |  | Unknown | | 0.529 |
|  | p.Glu696Lys | TOL | 0.08 |  | |  | Unknown | | 0.841 |
| NM_018965.3 | p.Arg47His | TOL | 0.11 | 5ELI | | -1.120 | Unknown | | 0.338 |
|  | p.Thr66Met | DEL | 0 | 5ELI | | -1.116 | Unknown | | 0.320 |
| NM_000371.3 | p.Val50Met | DEL | 0.03 | 1F64 | | -0.568 | Unknown | | 0.663 |
|  | p.Ile127Val | TOL | 0.55 | 1F64 | | -0.228 | Unknown | | 0.553 |
| NM_004738.4 | p.Pro56Ser | DEL | 0.01 | 2MDK | | -1.713 | Pathogenic | | 0.968 |
| NM_007126.3 | p.Asn91Tyr | DEL | 0 | 3QC8 | | -1.123 | Pathogenic | | 0.961 |
|  | p.Arg155Cys | DEL | 0.01 | 3QC8 | | -0.973 | Pathogenic | | 0.994 |
|  | p.Arg155His | TOL | 0.06 | 3QC8 | | -1.080 | Pathogenic | | 0.976 |
|  | p.Arg159Cys | DEL | 0 | 3QC8 | | -0.665 | Pathogenic | | 0.992 |
|  | p.Ala232Glu | TOL | 0.23 | 3QC8 | | -0.689 | Pathogenic | | 0.977 |

## Gene Ontology (GO) enrichment analysis for 289 genes

We performed GO enrichment analysis on the 289 genes which were included in our database. As shown in Figure S1, those genes were significantly enriched in 25 GO terms (p<0.05 and FDR <0.05), including 11 biological process terms, 11 cellular component terms and 3 molecular function terms. Most of the terms play important roles in neurodegenerative disease. For example, numerous studies have demonstrated the oxidative stress had been consistently linked to ageing-related neurodegenerative disease and induced apoptosis and protein misfolding in neurons (1,2). Since endoplasmic reticulum (ER) is an important organelle for neuronal survival and function and sensitive to alterations in cellular homeostasis, the ER stress also serves as an important role in neuron apoptosis (3,4).


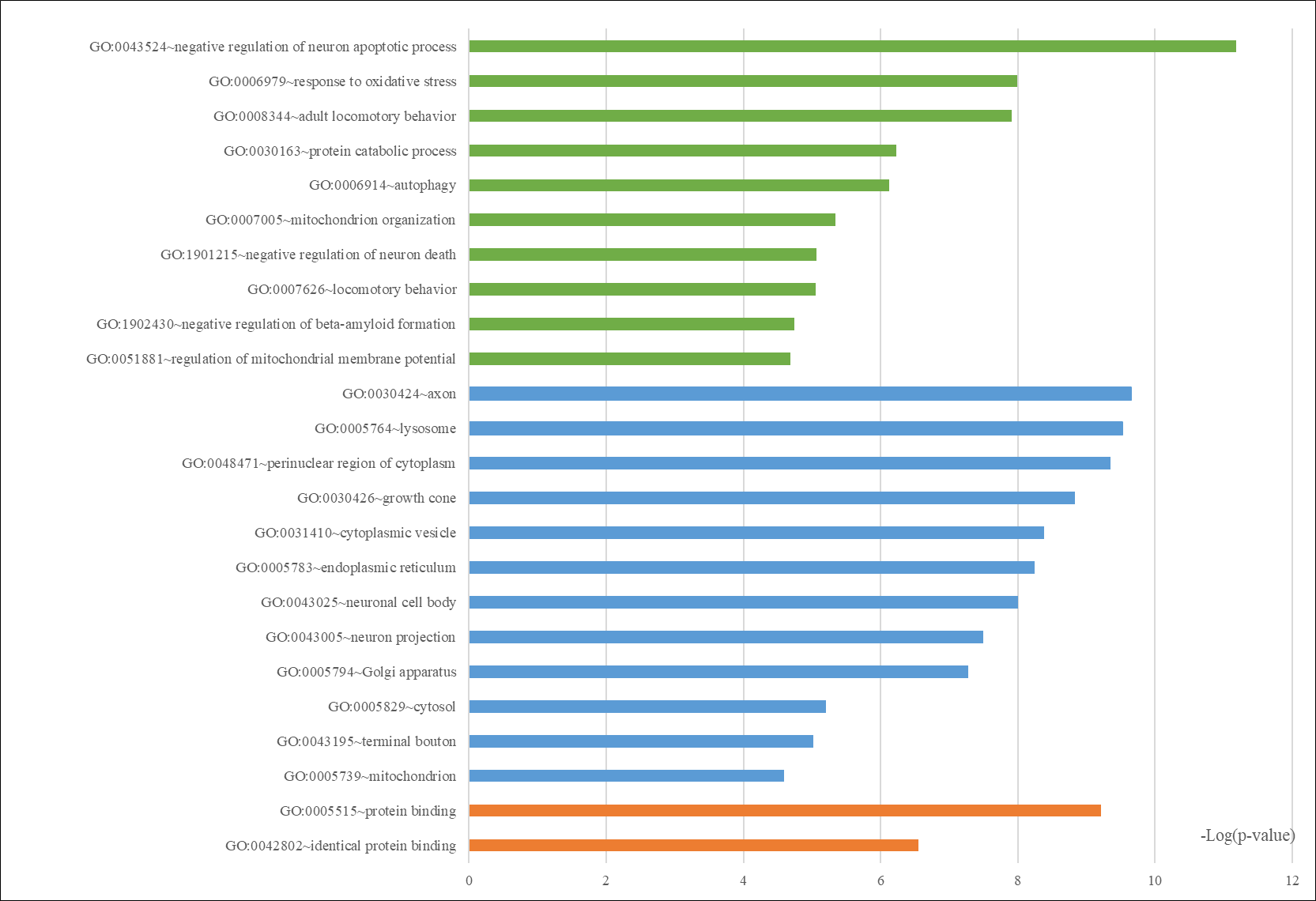
Supplementary figure 2. GO enrichment analysis. The enriched terms belong to biological process, cellular component and molecular function were labeled as green, blue and orange, separately.

References

1. Ghosh, N., Ghosh, R., Mandal, S.C. (2011) Antioxidant protection: A promising therapeutic intervention in neurodegenerative disease. *Free Radic Res*, **45**, 888-905.

2. Halliwell, B. (2006) Oxidative stress and neurodegeneration: where are we now? *J Neurochem*, **97**, 1634-1658.

3. Ye, J., Han, Y., Chen, X.*, et al.* (2014) L-carnitine attenuates H2O2-induced neuron apoptosis via inhibition of endoplasmic reticulum stress. *Neurochem Int*, **78**, 86-95.

4. Logue, S.E., Cleary, P., Saveljeva, S.*, et al.* (2013) New directions in ER stress-induced cell death. *Apoptosis*, **18**, 537-546.
